# The Debut Signal of Bone Metastasis and Stealthy Gastric Cancer Unmasked in a Young Male: A Case Report

**DOI:** 10.7759/cureus.61421

**Published:** 2024-05-31

**Authors:** Parav Tantia, Abhinav Kadam, Jagrati Yadav, Sourya Acharya, Sunil Kumar

**Affiliations:** 1 Department of Medicine, Jawaharlal Nehru Medical College, Datta Meghe Institute of Medical Sciences (Deemed to be University), Wardha, IND; 2 Department of Medical Oncology, Jawaharlal Nehru Medical College, Datta Meghe Institute of Medical Sciences (Deemed to be University), Wardha, IND

**Keywords:** pet scan, stomach cancer, neoplasm, bony secondaries, carcinoma

## Abstract

Gastric cancer is the fifth leading cause of cancer-related deaths in the world. The occurrence of bone metastases (BM) in gastric cancer without prior gastrointestinal (GI) symptoms is a rare phenomenon that has been sporadically documented in the existing literature. We report a case of a 27-year-old male presenting with chief complaints of severe backache for one month.

After an upper gastrointestinal endoscopy and biopsy, the primary source of cancer was identified as a solitary gastric adenocarcinoma, supporting the diagnosis of bony metastases on the magnetic resonance imaging (MRI) of the spine. The patient was planned to start on palliative chemotherapy (5-fluorouracil, leucovorin, oxaliplatin, and docetaxel {FLOT} regimen) with palliative radiotherapy of 20 Gy in five fractions to bony metastasis. The patient denied treatment and was discharged against medical advice.

## Introduction

Mesenchymal tissue cancers are frequently compounded by metastasis to different distant tissues, and they most frequently disseminate hematogenously. Malignancies of the prostate, breast, lung, kidney, bladder, and thyroid usually spread to the bone. Rarely may cancer of the gastrointestinal (GI) tract spread to the bone. Rarely, disseminated bone tumors appear as the earliest clinical signs; these cases have been reported to have a very bad prognosis.

Males were more likely than females (39.5%) to develop GI malignancies (60.5%). Cancer of the esophagus was the most prevalent type among males (28.2%), with stomach and rectum cancers following closely behind (21% and 14.3%, respectively). Cancers of the stomach (14.8%), rectum (14.6%), gallbladder (23.8%), and esophagus (25.7%) were prevalent in females. The most prevalent kind of GI system cancer was adenocarcinoma (57.83%), and the second most common is squamous cell neoplasms (25.99%). Nearly 5%-15% of the patients with gastric carcinoma are aged <40 years, and only 1%-2% of the patients are aged <30 years. The northeastern region of India's Aizawl district (age-adjusted rate {AAR}: 126.9 for males) and Papumpare district (AAR: 75.9 for females) had the greatest prevalence of GI cancer in India. Gastric carcinoma is rare in young people; it commonly shows a more aggressive biological behavior with a worse prognostic result. The majority of gastrointestinal malignancies manifested at the locoregional stage [1]. It is atypical and not often documented in the literature for stomach cancer to manifest as bone metastases (BM) without any prior gastrointestinal symptoms. The incidence of bone metastasis in GI carcinoma was 3.8% [2], and the presentation of bone secondaries prior to gastric cancer manifestation was found to be even rarer.

In this article, we present a case of gastric cancer that initially presented with trivial complaints such as lower back pain, which was found to have bony metastasis of the lumbosacral spine. Upon further investigation, the patient was found to have a neoplastic lesion in the stomach, which was confirmed as an adenocarcinoma on a biopsy.

## Case presentation

**Figure 1:**
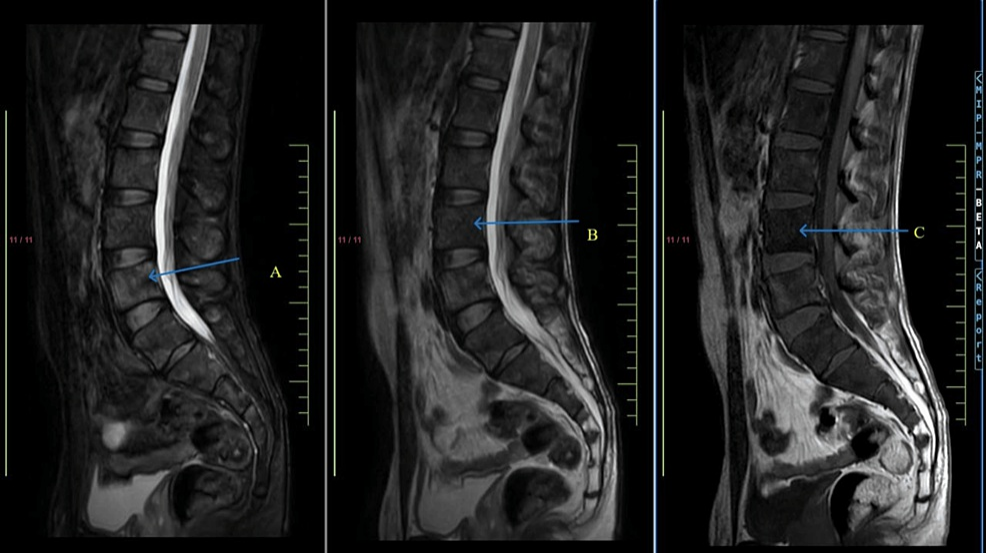
Magnetic resonance imaging (MRI) of the whole spine of the patient suggestive of depression at D4 with a slightly convex posterior margin abutting the ventral cord surface without canal stenosis. (A) Heterogenous hyperintensity in T2 FS, (B) diffuse T2 hypointensity in T2 image, and (C) diffuse T1 hypointensity in T1 image. Diffuse T1 hypointense signals in vertebrae with heterogeneous hyperintensity on STIR with multiple rounded STIR hyperintense lesions within and similar marrow signal changes and lesions in the pelvic bones, sacrum, and proximal femora. There was 20% superior endplate depression at D4 with a slightly convex posterior margin abutting the ventral cord surface without canal stenosis. D6, D11, and D12 vertebral bodies. Also, slightly convex posterior margins abutting the ventral cord surface without canal stenosis were shown. Thirty percent compression deformity at L1. No posterior retropulsion or canal stenosis. FS, fat-suppressed; STIR, short tau inversion recovery

**Figure 2:**
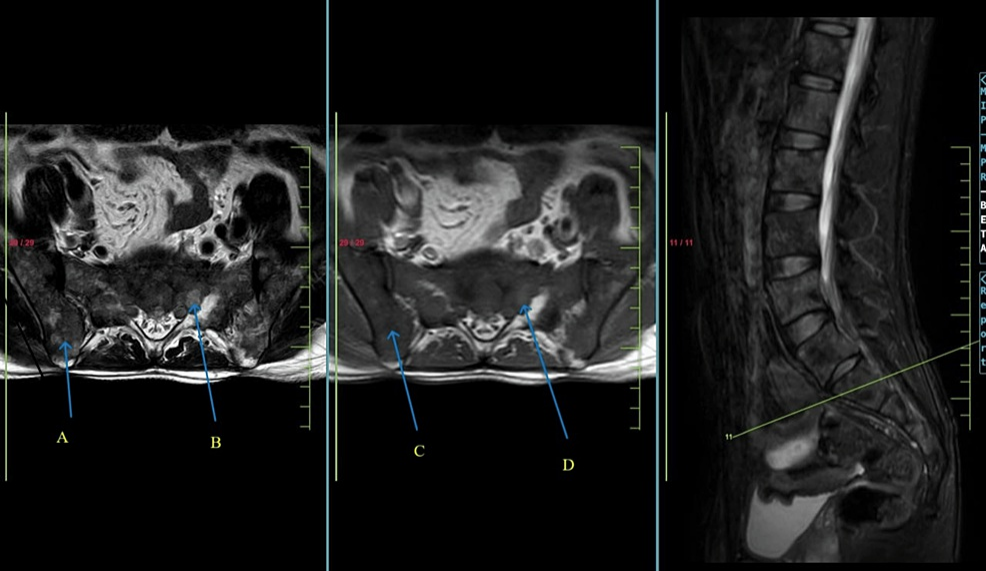
Magnetic resonance imaging (MRI) of the whole spine of the patient suggestive of minimal anterolisthesis of L5 over S1 with bilateral L5 pars interarticularis defects. (A) Pelvic bone heterogenous signal intensity in the T2 axial image, (B) sacrum bone heterogenous signal intensity in the T2 axial image, (C) pelvic bone hypointensity in the T1 axial image, and (D) sacrum bone hypointensity in the T1 axial image.

A 27-year-old male presented to the outpatient department with complaints of acute backache for one month, which radiated to the lower back, and the patient was not able to walk due to excruciating pain that started after injury while doing strenuous exercise (weight lifting). The patient stated a history of visiting a private practitioner for the same, where he was treated symptomatically and was given painkiller injectables and sent home on an outpatient basis. He was then feeling comfortable in the morning and resumed his daily activity. He then complained of similar episodes of backache on multiple occasions, following which he came to this hospital after one month of initial symptoms. Based on symptoms and examination, he was advised to get a magnetic resonance imaging (MRI) of the whole spine done, which was suggestive of depression at D4 with a slightly convex posterior margin abutting the ventral cord surface without canal stenosis (as shown in Figure 1) and minimal anterolisthesis of L5 over S1 with bilateral L5 pars interarticularis defects (as shown in Figure 2).

The findings on MRI were suggestive of neoplasm (metastasis or leukemia); hence, further evaluation is required. For further evaluation, the patient was advised to get a positron emission tomography (PET) scan done.

**Figure 3:**
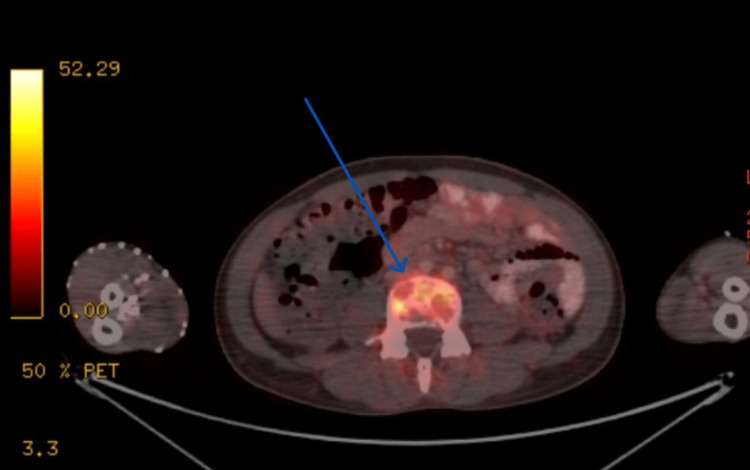
Positron emission tomography (PET) scan of the patient is suggestive of hypermetabolic extensive lytic skeletal lesions with soft tissue components with marrow involvement throughout the visualized axial. Hypermetabolic extensive lytic skeletal lesions with soft tissue component with marrow involvement throughout the visualized axial and appendicular skeletal system and metabolically active gastro-hepatic node. Mild mucosal thickening with low-grade metabolism involving the proximal two-thirds of the body of the stomach appears to be inflammatory in etiology. There is no obvious e/o metabolic disease elsewhere to comment upon the site of primary malignant disease etiology. e/o: evidence of

**Figure 4:**
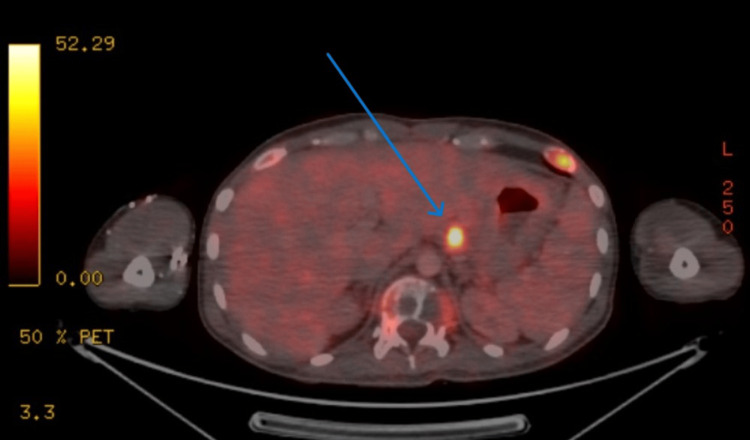
Positron emission tomography (PET) scan of the patient is suggestive of metabolically active gastro-hepatic node. Mild mucosal thickening with low-grade metabolism involving the proximal two-thirds of the body of the stomach.

The PET scan was suggestive of hypermetabolic extensive lytic skeletal lesions with soft tissue components with marrow involvement throughout the visualized axial and appendicular skeletal system (Figure 3) and metabolically active gastro-hepatic node (Figure 4). There is no obvious evidence of (e/o) metabolic disease elsewhere to comment upon the site of primary malignant disease etiology.

As the patient presented with only symptoms of pain and radio imaging was suggestive of metastasis, the primary cause could not be found. Henceforth, based on symptoms and history, the patient was suspected of having multiple myeloma, and hence, bone marrow aspiration was done.

Bone marrow aspirate was suggestive of erythroid hyperplasia with micro-normoblastic erythropoiesis. Hence, there was no evidence for this diagnosis.

He was advised blood investigations for further evaluation. CA 19-9 level was raised suggestive of malignancy of the GI tract. Vitamin D values were low, which could be due to bony lytic activity as shown in Table 1.

**Table 1:** Laboratory parameters of the patient. Hb, hemoglobin; INR, international normalized ratio; CEA, carcinoembryonic antigen; AST, aspartate aminotransferase; ALT, alanine aminotransferase; ALP, alkaline phosphatase

Test	Value	Normal Values
Hb	7.8 g/dL	11-14 g/dL
WBC	8,200 cells/cumm	4,000-11,000 cells/cumm
Platelet	0.93 cells/cumm	1.50-4.50 x 10^6 ^cells/cumm
Total RBC count	2.97 cells/cumm	2.50-5.50 x 10^6 ^cells/cumm
INR	1.32	1.00
CA 19-9	80.4 µ/mL	<37 µ/mL
CEA	2.93 ngm/mL	<3 ngm/mL
Urea	50 mg/dL	9-20 mg/dL
Creatinine	1.1 mg/dL	0.66-1.25 mg/dL
Sodium	136 mmol/L	135-145 mmol/L
Potassium	4.0 mmol/L	3.5-5.1 mmol/L
AST	40 U/L	<50 U/L
ALT	17 U/L	<50 U/L
ALP	481 U/L	38-126 U/L
Albumin	3.7 g/dL	3.5-5 g/dL
Total bilirubin	0.4 mg/dL	0.2-1.3 mg/dL
Vitamin D	16.9 ngm/mL	30-100 ngm/mL
Urine culture	No growth	
Blood culture	No growth	

**Figure 5:**
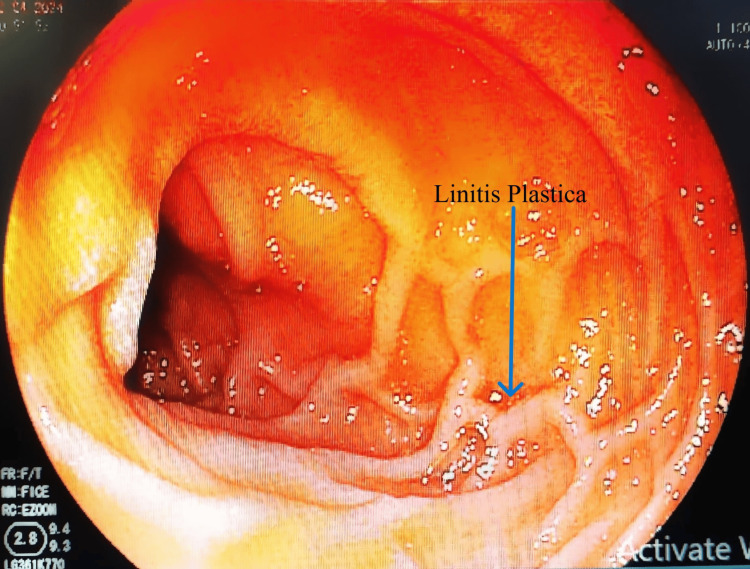
Upper GI endoscopy suggestive of stasis esophagitis with linitis plastica pattern. Multiple biopsies were taken from the same site. GI: gastrointestinal

On further evaluation, a gastroenterology opinion was taken based on all the above findings and investigation; upper GI endoscopy was advised given the presence of mild mucosal thickening and metabolic active gastro-hepatic nodes on the PET scan. Upper GI endoscopy was suggestive of stasis esophagitis with linitis plastica (Figure 5). Multiple biopsies were taken from the same site and sent for histopathological study.

**Figure 6:**
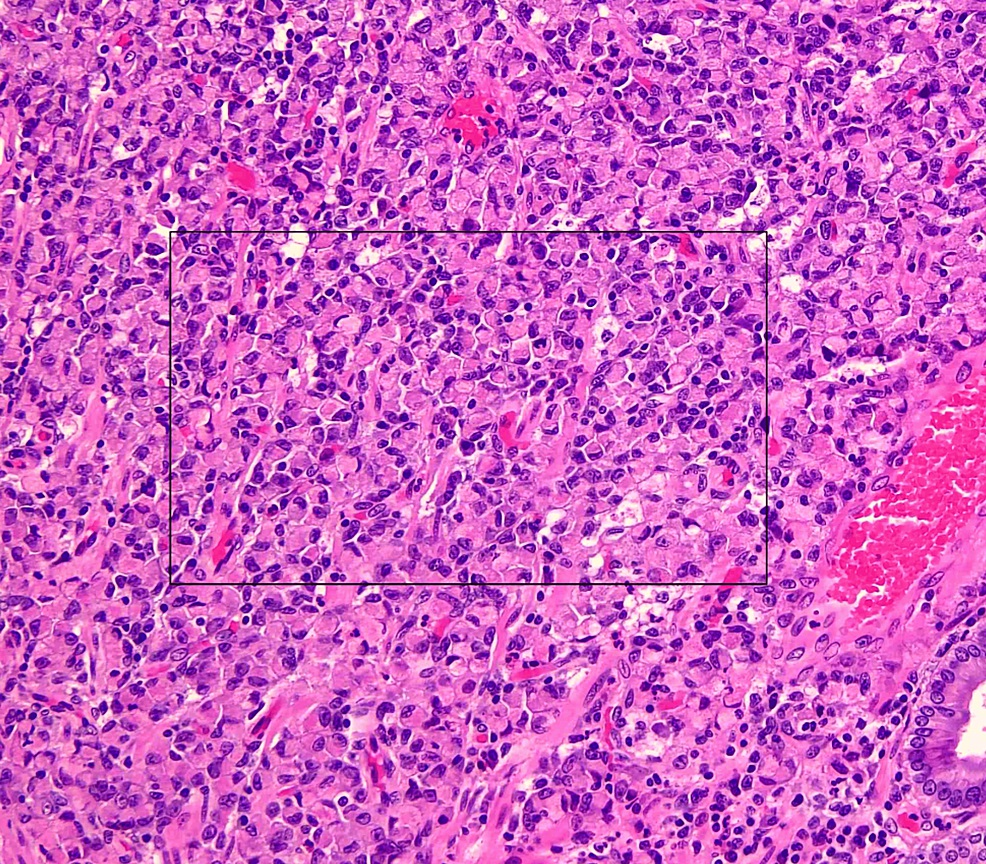
Section from the stomach showing areas predominantly composed of signet ring cells (black box), which are characteristically optically clear, central, globoid droplets of cytoplasmic mucin with an eccentric nucleus (H&E: 40×).

On the biopsy report, it was found that the mass from the gastrointestinal tract was suggestive of adenocarcinoma (signet ring type) (Figure 6).

The patient was treated on the basis of symptoms, and palliative treatment was given. After the histopathological report, the patient's diagnosis was made to be adenocarcinoma of the stomach with bony metastasis.

The patient was planned to start on palliative chemotherapy (5-fluorouracil, leucovorin, oxaliplatin, and docetaxel {FLOT} regimen) with palliative radiotherapy of 20 Gy in five fractions to bony metastasis: 5-fluorouracil (5-FU), 2,600 mg/m^2^ IV continuous infusion over 24 hours on day 1; leucovorin, 200 mg/m^2^ IV on day 1; oxaliplatin, 85 mg/m^2^ IV on day 1; and docetaxel 50 mg/m^2^ IV on day 1, with a plan of a minimum of five cycles followed by assessment for further dosage.

As the patient and relatives were not willing for chemotherapy and radiotherapy, palliative chemoradiotherapy was not taken by the patient. Moreover, they took discharge against medical advice.

## Discussion

On a global scale, gastric cancer continues to be listed as the fifth most common cause of cancer-associated deaths [3]. It can spread to the spleen, adrenal gland, ovary, lung, brain, and skin, with usual metastases to the liver, lymph nodes, and peritoneal membrane [4]. Patients with stomach cancer have quite rare instances of bone metastases, which is indicative of a poor prognosis [5]. The mean survival period for stomach cancer patients with bone metastases is 4-5 months [6]. Furthermore, there are very few reports in the literature of gastric cancers that present as bone metastases in the absence of any gastrointestinal symptoms beforehand [7].

Yoshikawa and Kitaoka [8] reported that the use of radiation therapy effectively reduced bone pain in 23 individuals diagnosed with stomach cancer and bone metastases.

Serum alkaline phosphatase (ALP) levels in patients with bone metastasis of recurrent gastric cancer were found to be relatively related to the condition of bone metastasis (mildly increased). The prognosis of patients with bone metastasis was not good, and the mean survival time was about five months after the appearance of symptoms [9].

The results of both studies are mentioned in Table 2.

**Table 2:** Various studies on gastric carcinoma with bony metastasis.

Author	Title	Result
Yoshikawa and Kitaoka [8]	Bone metastasis of gastric cancer	1%-20% of cases have bone metastases
Nishidoi and Koga [9]	Clinicopathological study of gastric cancer with bone metastasis	13.4% of the 246 individuals with stomach cancer had bone metastases

In cases of poorly differentiated adenocarcinoma, signet ring cell carcinoma, in relatively younger age, diffusely involved stomach cancer or a Borrmann type 4 morphology; along with cases with abundant lymph node metastasis nearby, bone metastasis may occur more frequently [10].

Cancer multiplying widely in the bone marrow can lead to disseminated carcinomatosis [11]. When they multiply quickly, bone deterioration and hematological problems additionally develop [12].

It is unknown how precisely tumor cells spread to the bone. According to Lehnert et al. [13], the abundance of blood capillaries in the stomach mucosa may have a role in the cancer's early bone metastasis.

It is also proposed that bone metastasis from stomach carcinoma may occur via a different, non-portal pathway via the vertebral venous plexus. Bone scintigraphy was able to identify bone metastases about three months ahead of time when utilizing standard X-ray technology [14].

However, due to their low specificity, hot uptake lesions can be mistaken for various other lesions, including degenerative arthritis, fractures, infectious bone disorders, benign bone diseases, Paget's disease, and primary bone cancers. Additional positron emission tomography-computed tomography, bone marrow tapping, magnetic resonance imaging, or bone marrow histology testing carried out in parallel may further improve the diagnosis accuracy.

According to multiple studies, bone metastasis most commonly occurs in the vertebrae [15]. These results confirm that the primary pathway for bone metastases is the vertebral venous system. According to Choi et al. [15], the vertebrae (66%), pelvic bone (43%), costa (59%), femur (30%), and scapula and clavicle (17%) were the most common sites of metastasis. Pathologic fractures, bone pain, and spinal cord compression are the most prevalent clinical signs and symptoms of bone metastases. Elevated alkaline phosphatase (ALP), elevated lactate dehydrogenase (LDH), and anemia or thrombocytopenia are among the laboratory abnormalities that may indicate the presence of bone metastases.

In a related study, McQuay et al. [16] noted that after one month of receiving radiation treatment, a quarter of the patients experienced complete relief from bone pain. At the same time, a third of them achieved a minimum of 50% reduction in pain levels.

When treating advanced metastatic gastric carcinomas, chemotherapy may improve quality of life more than symptomatic care alone. According to earlier research, the majority of patients with early BM improved dramatically and quickly following chemotherapy in terms of Eastern Cooperative Oncology Group performance status (ECOG PS), hematological abnormalities, and ALP levels [17].

Treatment with S1+paclitaxel, methotrexate (MTX)+5-FU, S1+cisplatin, and other chemotherapy recently extended the survival duration. Clinical symptoms resulting from problems and bone metastases have been treated with bisphosphonates. Through the inhibition of bone reabsorption, it inhibited the synthesis of proliferative factors by the bones, which in turn inhibited the growth of cancer cells. However, additional research appears to be required to validate these findings [18].

## Conclusions

In summary, the case of gastric carcinoma with bony metastasis in a young male highlights the devastating impact of advanced-stage cancer.

The suspicion of gastric carcinoma on incidental bony lesions and vice versa, as well as looking for bony metastasis in gastric carcinoma, is a crucial step during the management of patients. This case highlights the varied presentation of gastrointestinal carcinoma and the importance of prompt diagnosis by physicians to detect and diagnose such scenarios. As in this case, young individuals with such presentation warrant a greater degree of vigilance among clinicians as misdiagnosis can lead to devastating effects.
